# Effects of ipsilateral tilt position on the cross-sectional area of the subclavian vein and the clinical performance of subclavian vein catheterization: a prospective randomized trial

**DOI:** 10.1186/s12871-020-01144-1

**Published:** 2020-09-05

**Authors:** Hyun-Kyu Yoon, Hyung-Chul Lee, Pyoyoon Kang, Jung-Man Lee, Hee-Pyoung Park, Youn Joung Cho

**Affiliations:** 1 Department of Anesthesiology and Pain Medicine, Seoul National University Hospital, Seoul National University College of Medicine, 101 Daehakro, Jongno-gu, Seoul, 03080 South Korea; 2 Department of Anesthesiology and Pain Medicine, SMG-SNU Boramae Medical Center, Seoul, South Korea

**Keywords:** Catheterization, Complications, Punctures, Subclavian vein, Supine position

## Abstract

**Background:**

The cross-sectional area of the subclavian vein (csSCV) is a crucial factor in the successful catheterization of the subclavian vein. This randomized controlled study investigated the effects of the csSCV on landmark-based subclavian vein catheterization.

**Methods:**

This study was performed using a two-stage protocol. During stage I, the csSCV was measured in 17 patients placed in the supine, 20° ipsilateral tilt, and 20° contralateral tilt positions in a random order. During stage II, landmark-based subclavian vein catheterization was randomly performed in patients placed in either the supine (group S, *n* = 107) or the ipsilateral tilt (group I, *n* = 109) position. The primary outcome measure was the csSCV in stage I and the primary venipuncture success rate in stage II. Secondary outcome measures were the time to successful venipuncture, the total catheterization time, the first-pass success rate, and the incidence of mechanical complications during catheterization.

**Results:**

The csSCV was significantly larger in the ipsilateral tilt than in either the supine or contralateral tilt position (1.01 ± 0.35 vs. 0.84 ± 0.32 and 0.51 ± 0.26 cm^2^, *P* = .006 and < .001, respectively). The primary venipuncture success rate did not differ significantly between the group S and I (57.0 vs. 64.2%, *P* = .344). There were also no significant differences in the secondary outcome measures of the two groups.

**Conclusions:**

The csSCV was significantly larger in patients placed in the ipsilateral tilt than in the supine position, but the difference did not result in better clinical performance of landmark-based subclavian vein catheterization.

**Trial registration:**

NCT03296735 for stage I (ClinicalTrials.gov, September 28, 2017) and NCT03303274 for stage II (ClinicalTrials.gov, October 6, 2017).

## Background

Central venous catheterization may be mandatory in the management of critically ill patients for various purposes [1, 2]. Although the subclavian vein is a preferred site of central venous catheterization due to lower rates of infection and thrombosis than the femoral vein [3, 4] or internal jugular vein [1, 4–7], mechanical complications such as arterial puncture, hematoma formation, inadvertent pneumothorax, and misplacement of the catheter tip during subclavian vein catheterization have been reported [8].

Although ultrasonography is widely used during vascular access in most medical facilities, and is recommended as a standard method during central venous catheterization by a number of professional organizations [9–11], a traditional landmark-based technique is still important and useful in certain clinical situation where the ultrasonography is not promptly available or when the operator is not familiar with the equipment. For anatomical landmark-based technique, although it is still uncertain how much increase in the cross-sectional area of the vein will affect the success of catheter placement, the cross-sectional area of the vein can theoretically have an impact on the success rate of venous catheterization by affecting the venipuncture success rate [12]. Previously, the cross-sectional area of the subclavian vein (csSCV) was shown to be affected by head, shoulder, or arm positioning [13–18], as well as by changes in intrathoracic pressure during mechanical ventilation [12]. However, the effects of the ipsilateral tilt position of the patient on the csSCV have yet to be investigated. In the ipsilateral tilt position, the cross-sectional area of the dependent subclavian vein may increase because of the hindered venous flow to the heart. Therefore, we hypothesized: (1) that the csSCV would be larger in patients placed in the ipsilateral tilt than in either the supine or the contralateral tilt position, and (2) that, compared with the supine position, the ipsilateral tilt position would improve the primary venipuncture success rate by increasing the csSCV during catheterization.

In this study, we aimed to investigate the effects of ipsilateral tilt position on both the csSCV and the clinical performance of landmark-based subclavian vein catheterization. Therefore, we compared the csSCV among three different positions and the primary venipuncture success rate in the ipsilateral tilt versus supine position. Additionally, the clinical performance of subclavian vein catheterization in the two positions was determined by investigating the time to successful venipuncture, the total catheterization time, the first-pass success rate, and the incidence of mechanical complications during subclavian vein catheterization.

## Methods

### Study populations

After the approval of the institutional review board of Seoul National University Hospital (1707–110-871, Seoul, Korea) was obtained, the study protocols were registered at ClinicalTrials.gov on September 28, 2017 (NCT03296735 for stage I) and on October 6, 2017 (NCT03303274 for stage II) prior to enrollment, and published [19]. Written informed consent was obtained from all patients before enrollment. This study was performed under Good Clinical Practice Guidelines and adhered to the applicable Consolidated Standards of Reporting Trials (CONSORT) guidelines. Patients between the ages of 20 and 80 years, with ASA physical status classification I–III, and undergoing brain tumor surgery requiring subclavian vein catheterization between November 1, 2017 and August 31, 2018 were enrolled in the study. Patients who refused participation and who were not suitable for subclavian vein catheterization due to infection at the puncture site, tumor or thrombus along the course of the subclavian vein, or anticoagulation treatment were excluded from the study. Patients with a pacemaker or chemoport in the subclavian vein and patients who had previous breast cancer surgery or pneumonectomy were also excluded.

**Fig. 1:**
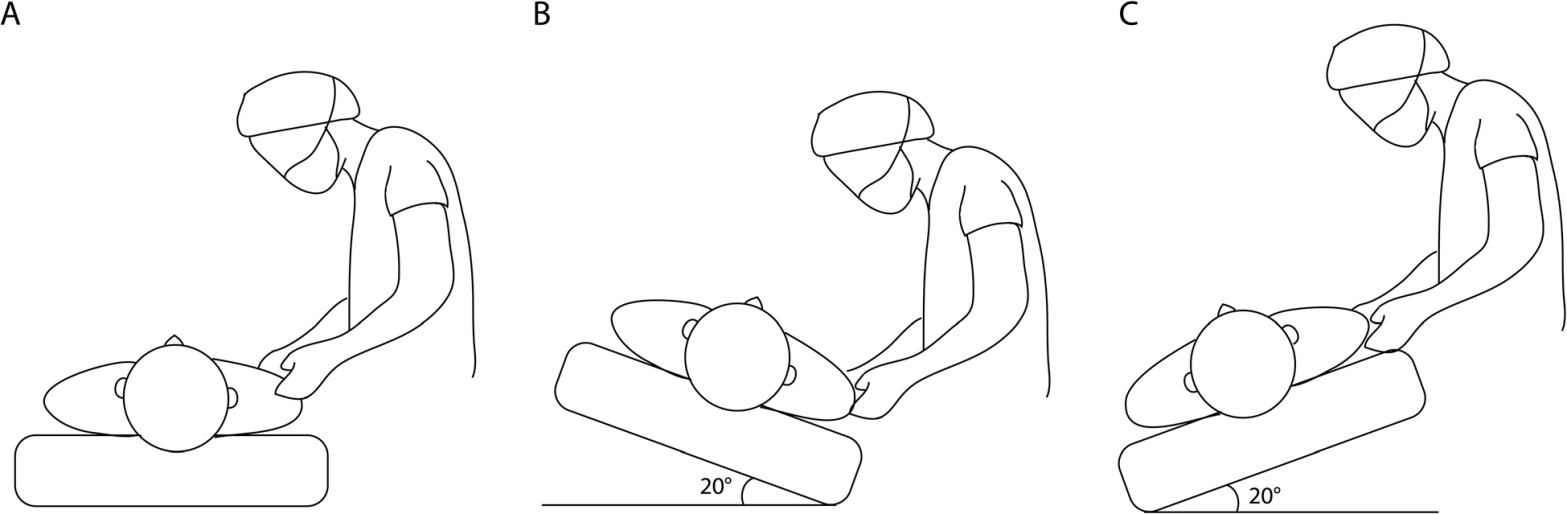
In stage I, the patients were placed in all three different positions without Trendelenburg positioning, but in a random order: **a** the supine position, **b** the ipsilateral tilt position with a 20° angle, **c** the contralateral tilt position with a 20° angle

This study was performed using a two-stage protocol. In stage I, which had a crossover design, after anesthetic induction, the patients were placed in all three different positions without Trendelenburg positioning, but in a random order: supine, ipsilateral tilt with a 20° angle, or contralateral tilt with a 20° angle (Fig. 1). The tilt positions were achieved by tilting the operating table maximally. In each position, an assistant ensured that the head and neck of the patient were kept in a neutral position. Each of the three positions was maintained for at least 1 min before the csSCV was measured. A 4.5–8 MHz linear ultrasound probe (Vscan Extend; GE Vingmed ultrasound, Horten, Norway) was placed in the mid-portion of the clavicle and was positioned to be perpendicular to the long axis of the subclavian vein. The csSCV was scanned at the end of the expiration with the ultrasound probe and measured using the image processing software (ImageJ; National Institutes of Health, Bethesda, MD, USA).

### Randomization

During stage II, block randomization with a mixture of blocks of size four and six was performed by an independent investigator blinded to the group assignment. Patients were randomly allocated in a 1:1 ratio to either the supine group (group S) or the ipsilateral tilt group (group I) according to the randomization order. The patients, surgeons, and data analyzers were blinded to the group assignment. The allocation order was concealed in opaque envelopes and was disclosed by an investigator just before the anesthetic induction.

### Subclavian vein catheterization technique

Following the anesthetic induction and tracheal intubation, the landmark-based subclavian vein catheterization via infraclavicular approach was performed by one of the two anesthesiologists who performed > 50 successful infraclavicular landmark-based subclavian vein catheterization using the Seldinger technique. The catheterization process was as follows. After skin preparation with an antiseptic solution, aseptic drape was applied around the midpoint of the clavicle. Additional maneuvers which may affect the csSCV, such as pulling the patient’s arms or placing a shoulder roll under the scapulae, were not performed throughout the procedure. The patients were then placed in either the supine or ipsilateral tilt position according to the group allocation. Each position was maintained for at least 1 min before an introducer needle was inserted. In patients in the ipsilateral tilt position, flank and knee belts were securely strapped, and one of the assistants held the patient’s trunk to prevent from falling. Another assistant kept the patient’s head and neck in a neutral position during catheterization. The skin was punctured 1 cm caudally and laterally from the inferior border of the midpoint of the clavicle. The needle was first contacted with the clavicle, then it was advanced towards the suprasternal notch beneath the clavicle with creating negative pressure within the syringe attached to the introducer needle. After regurgitation of the blood had been confirmed, a needle attached to transducer was connected to the hole at the end of the syringe to rule out arterial puncture. In case of arterial puncture, the needle was removed and the bimanual pressure above and below the clavicle was applied to control hemorrhage for > 5 min. Following confirmation of successful venipuncture, a guidewire was introduced through the needle, and a dilator was used to facilitate the insertion of a 7-Fr double-lumen central venous catheter (Arrow International Inc., Reading, PA, USA) through the guidewire. Mechanical ventilation was stopped at the time of skin puncture and then restarted after catheter insertion. During catheterization, when the peripheral oxygen saturation was checked below 94% during interruption of ventilation, catheterization was halted and rescue ventilation was provided until the oxygen saturation ≥ 95%.

If the first attempt of venipuncture failed, the needle was withdrawn slowly to the level of the subcutaneous tissue and then redirected according to the discretion of the attending anesthesiologist. A maximum of three attempts per operator was allowed. If two operators failed to achieve successful venipuncture after a total of six attempts, either the internal jugular or femoral vein was selected for catheterization. The number of venipuncture attempts and the time to successful venipuncture were recorded during catheterization. The number of attempts required for successful guidewire, dilator, and catheter insertion was also recorded. Total catheterization time, defined as the interval between skin puncture and catheter placement, was recorded as well. All catheterization-related parameters were recorded by an anesthesia nurse who did not know about the study. For evaluation of catheterization-related mechanical complications, the chest radiography was taken in all patients after the procedure, and the ultrasonography was checked if necessary.

### Study outcomes

The primary outcome measure was the csSCV in stage I and the primary venipuncture success rate in stage II. Secondary outcomes were the incidence of mechanical complications, including arterial puncture, subcutaneous hematoma formation, inadvertent pneumothorax, and misplacement of the catheter tip (indwelling of the catheter tip other than in the right atrium or superior vena cava); the number of venipuncture attempts; the number of attempts required for successful guidewire, dilator, and catheter insertion; the first-pass success rate of the catheterization which was defined as when all steps of catheterization from venipuncture to catheter insertion were successful at the first attempt; the time to successful venipuncture; and the total catheterization time.

### Sample size determination

Sample size was determined according to the stage of the study. In a previous study, the mean ± SD of the csSCV measured in the supine position was 0.93 ± 0.17 cm^2^ [14]. To obtain a 15% increase in the csSCV of the same patient placed in the ipsilateral tilt versus the supine position, the enrollment of 15 patients was needed to achieve a two-tailed level of significance of 0.017 (0.05/3) and a power of 80%. Considering a 10% dropout rate, 17 patients were enrolled in stage I of the study. A previous study showed that the primary venipuncture success rate was 74.5% during subclavian vein catheterization of patients in the supine position [20]. Thus, in stage II of this study, the achievement of a 15% increase in the primary venipuncture success rate in patients placed in the ipsilateral tilt versus the supine position required the enrollment of 100 patients per group, based on a significance level of 0.05 and a power of 80%. Considering a 10% dropout rate, 110 patients per group were enrolled.

### Statistical analysis

The csSCV of patients placed in the three positions during stage I were compared using the Wilcoxon signed rank test with an alpha of 0.017 following a Bonferroni correction for multiple comparisons. The continuous variables of patients in stage II were compared using Student’s *t*-test or the Mann-Whitney *U* test according to the results of the Kolmogorov-Smirnov test. Discrete variables were analyzed using the chi-squared test or Fisher’s exact test. SPSS software (version 25.0; IBM Corp., Armonk, NY, USA) was used for all statistical analyses. A *P* value < .05 was considered to indicate statistical significance.

## Results

**Fig. 2:**
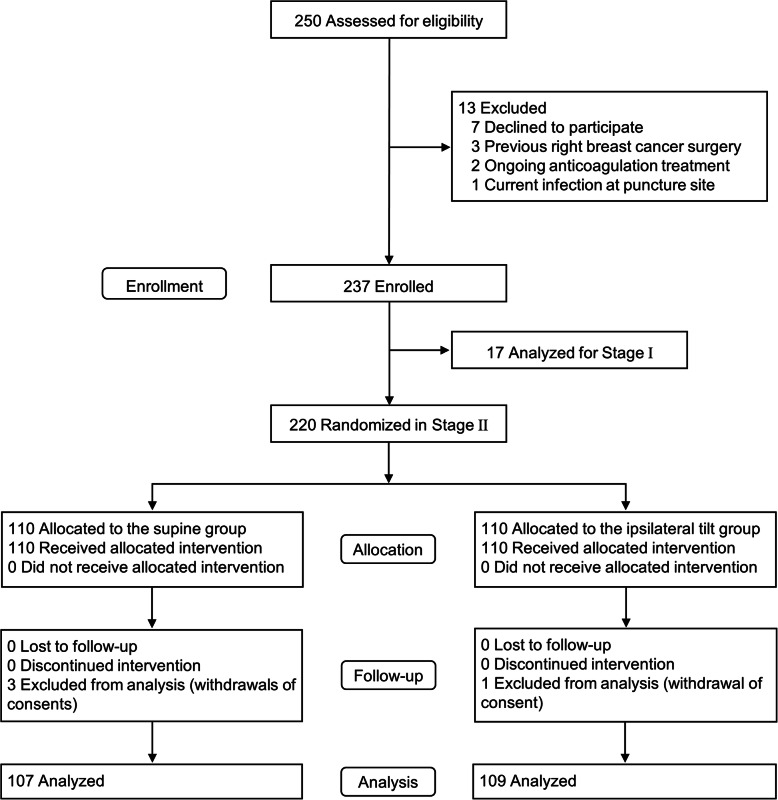
CONSORT flowchart

Of the 250 patients eligible for the study, 13 were excluded. The remaining 237 patients (17 patients in stage I and 220 patients in stage II) were enrolled in the study (Fig. 2). Four patients in stage II withdrew their consent, such that the final analyses were based on the data of 17 patients in stage I and 216 patients in stage II. The baseline characteristics of the included patients are presented in Table 1.

**Table 1:** Patient characteristics of enrolled patients in stage I and II

Characteristics	Stage I (*n* = 17)	Stage II	
		Group S (*n* = 107)	Group I (*n* = 109)
Age (years)	52 (37–64)	55 (42–61)	55 (43–64)
Gender			
Male	9 (52.9)	47 (43.9)	56 (51.4)
Female	8 (47.1)	60 (56.1)	53 (48.6)
Height (cm)	162.9 ± 9.5	163.7 ± 9.6	162.5 ± 9.4
Weight (kg)	66.7 ± 12.9	61.4 ± 11.1	63.5 ± 12.7
BMI (kg/m^2^)	24.6 ± 3.3	22.9 ± 3.4	23.9 ± 3.6
ASA PS			
I	7 (41.2)	39 (36.4)	30 (27.5)
II	10 (58.8)	53 (49.5)	64 (58.7)
III	0 (0.0)	15 (14.0)	15 (13.8)

Data are presented as median (interquartile range), number of patients (%), or mean ± SD

*BMI* body-mass index, *ASA PS* American Society of Anesthesiologists Physical Status classification

In the group S, landmark-based subclavian vein catheterization was performed in the supine position. In the group I, landmark-based subclavian vein catheterization was performed in the ipsilateral tilt position with a 20° angle

In stage I, the csSCV was significantly larger when patients were placed in the ipsilateral tilt than in the supine or contralateral tilt position (1.01 ± 0.35 vs. 0.84 ± 0.32 cm^2^ and 0.51 ± 0.26 cm^2^; *P* = .006 and < .001, respectively).

In stage II, there was no significant difference in the primary venipuncture success rate of groups S and I (57.0 vs. 64.2%, *P* = .344, Table 2). Venipuncture-related parameters, including time to successful venipuncture and the number of venipuncture attempts, did not differ between the two groups. The number of attempts required for successful dilator insertion was higher in patients in the I group than in the S group (1.3 ± 0.6 vs. 1.1 ± 0.3, *P* = .001). However, other catheterization-related variables, including the first-pass success rate of catheterization, the number of attempts needed for successful guidewire and catheter insertion, and the total catheterization time, were similar between the two groups. All procedure-related mechanical complications are presented in Table 3. There was no significant difference in the incidence of mechanical complications between the supine and ipsilateral tilt groups.

**Table 2:** Procedure-related variables in patients undergoing subclavian vein catheterization in the supine and ipsilateral tilt position

Parameters	Group S (*n* = 107)	Group I (*n* = 109)	Mean (95% CI) difference	*P* value
Venipuncture				
Primary venipuncture success rate	61 (57.0)	70 (64.2)	7.2% (− 5.8 to 19.8)	.344
Time to successful venipuncture (s)	21.9 ± 34.8	16.9 ± 24.9	5.0 (− 13.1 to 3.1)	.228
The number of venipuncture attempts (n)	2.4 ± 2.8	2.2 ± 3.1	0.2 (− 1.0 to 0.6)	.605
Successful venipuncture during the needle advancement	80 (74.8)	82 (75.2)	0.4% (−11.07 to 11.89)	.999
The number of attempts for insertion (n)				
Guidewire	1.4 ± 1.8	1.3 ± 0.9	0.1 (−0.5 to 0.3)	.361
Dilator	1.1 ± 0.3	1.3 ± 0.6	0.2 (0.1 to 0.3)	.001
Catheter	1.0 ± 0.3	1.0 ± 0.4	0.0 (− 0.1 to 0.1)	.988
Success rate of catheterization				
Overall	107 (100.0)	108 (99.1) ^*^	0.9% (−2.6 to 5.0)	.999
At the first attempt	92 (86.0)	97 (89.0)	3.0% (−6.0 to 12.1)	.643
At the second attempt	8 (7.5)	5 (4.6)	2.9% (−3.9 to 10.0)	.544
At the third attempt	5 (4.7)	6 (5.5)	0.8% (−5.7 to 7.4)	.999
At the fourth attempt	2 (1.9)	0 (0.0)	1.9% (−1.8 to 6.6)	.244
First-pass success rate	56 (52.3)	51 (46.8)	5.5% (−7.7 to 18.5)	.497
Total catheterization time (s)	106.6 ± 84.7	113.3 ± 80.7	6.7 (−15.5 to 28.9)	.551
The incidence of rescue ventilation	6 (5.6)	7 (6.4)	0.8% (−6.1 to 7.7)	.999

Data are presented as number of patients (%) or mean ± SD

*CI* confidence interval, *NA* not applicable

In the group S, landmark-based subclavian vein catheterization was performed in the supine position. In the group I, landmark-based subclavian vein catheterization was performed in the ipsilateral tilt position with a 20° angle. ^*^ In one patient in the group I, central venous catheter was placed on the right internal jugular vein after failed subclavian vein catheterization

**Table 3:** Procedure-related complications in patients undergoing subclavian vein catheterization in the supine and ipsilateral tilt positions

	Group S (*n* = 107)	Group I (*n* = 109)	Mean (95% CI) difference	*P* value
Total mechanical complications	10 (9.3)	7 (6.4)	2.9% (−4.6 to 10.6)	.586
Arterial puncture	1 (0.9)	0 (0.0)	0.9% (−2.6 to 5.0)	.495
Subcutaneous hematoma formation	2 (1.9)	4 (3.7)	1.8% (−3.4 to 7.4)	.683
Inadvertent pneumothorax	0 (0.0)	0 (0.0)	NA	NA
Misplacement of the catheter tip	9 (8.4)	3 (2.8)	5.6% (−0.8 to 12.6)	.081
Ipsilateral internal jugular vein	7 (6.5)	2 (1.8)	4.7% (−1.0 to 11.2)	.100
Contralateral innominate vein	2 (1.9)	1 (0.9)	1.0% (−3.3 to 5.8)	.620

Data are presented as number of patients (%)

*CI* confidence interval, *NA* not applicable

In the group S, landmark-based subclavian vein catheterization was performed in the supine position. In the group I, landmark-based subclavian vein catheterization was performed in the ipsilateral tilt position with a 20° angle

## Discussion

In this study, the csSCV was significantly larger when patients were placed in the ipsilateral tilt than in either the supine or contralateral tilt position. However, the primary venipuncture success rate during subclavian vein catheterization did not differ significantly between the patients in the supine and ipsilateral tilt groups. All catheterization-related parameters were comparable between the two positions, with the exception of the number of attempts for successful dilator insertion, which was higher in patients placed in the ipsilateral position.

Because the cross-sectional area of the vein is an important determinant of successful central venous catheterization, various maneuvers to increase the csSCV have been introduced. Among them, 30° head rotation to the ipsilateral side of the operator and arm positioning with 90° abduction, 90° flexion, and 90° external rotation have been shown to increase the csSCV [16, 18]. However, head rotation can disturb cerebral venous drainage, which may increase the intracranial pressure (ICP) [21], and specific arm positioning requires an additional device to maintain arm placement [18]. The Trendelenburg position increases the csSCV compared with the supine position [22], but it may increase the ICP, especially in patients with intracranial space-occupying lesions [23]. In our study, the csSCV increased by 16.7% when the patients were placed in the ipsilateral tilt position compared to the supine position. A previous study showed that there was no significant difference in ICP of neurocritical patients before and after the right lateral positioning [24].

For central venous catheterization, the subclavian vein is more preferred site than the femoral or internal jugular vein because it is less associated with thrombosis, infection, and patient discomfort during the maintenance of the catheter [1, 3–7, 25]. A meta-analysis reported that the first-pass success rate of subclavian vein catheterization using the landmark technique was 68.3% [26]. Moreover, an increased number of needling attempts during subclavian vein catheterization was associated with a higher rate of failure to catheterize and catheterization-related complications [8]. Therefore, the primary venipuncture success rate may be clinically related to the increased first-pass success rate of subclavian vein catheterization and may decrease catheterization-related complications. A recent study also suggested that an increase in the axillary vein area during mechanical ventilation may theoretically improve the first-pass success rate of central venous catheterization [12]. Accordingly, this study examined whether the primary venipuncture success rate could be improved by increasing the csSCV. However, our results showed that, although the csSCV increased significantly in the ipsilateral tilt versus the supine position, it did not improve the primary venipuncture success rate. These findings can be explained as follows. First, an increase in csSCV caused by positional change from the supine to the ipsilateral tilt position may not have been sufficient to result in a significant clinical impact on the primary venipuncture success rate. In our patients, the mean difference in csSCV between the two groups was 0.17 cm^2^, while statistically significant (*P* = .006), which might not have elicited a meaningful practical benefit. Second, in the landmark technique, anatomical relationships between the subclavian artery and vein may be a crucial factor in successful venipuncture at the first attempt. Generally, the subclavian vein is located anterior to the subclavian artery at the mid-clavicular point [27]. However, a previous study reported that in 36% of the evaluated patients the subclavian vein was located medial to the subclavian artery at the mid-clavicular site [28]. A medial or posterior position of the subclavian vein relative to the adjacent artery may decrease the primary venipuncture success rate during landmark-based subclavian vein catheterization.

In this study, there were no significant differences in the incidence of procedure-related mechanical complications in the groups S and I during landmark-based subclavian vein catheterization. The complication rates related to catheterization in both positions were 9.3 and 6.4%, respectively (*P* = .586). In previous studies, the incidence of subclavian vein catheterization-related complications was 5.8–16.8% [8, 20, 26, 29]. Moreover, the development of complications during subclavian vein catheterization has been associated with the number of needling attempts and the number of failed attempts at catheterization [8]. Another study reported a significant association between the duration of catheter insertion and the occurrence of catheterization-related mechanical complications [3]. In our patients, the total catheterization time and the number of venipuncture and failed catheterization attempts did not differ between the patients in the supine versus the ipsilateral tilt position.

According to our results, the number of attempts for dilator insertion was significantly higher in patients in the ipsilateral tilt than the supine position, presumably because the soft tissue of the patients would be shifted ipsilaterally to operator’s side by tilting the operating table in patients in the ipsilateral position. The use of excessive force to achieve dilator insertion and repeated attempts to insert the dilator can cause serious vessel injuries [30–32]. Moreover, although we did not observe any other significant difference in procedure-related variables between the groups, the ipsilateral position might negatively affect the number of needling or guidewire insertion as well. These can raise concerns about procedure-related safety issues related to the ipsilateral position. Therefore, additional caution would be required for patient safety during subclavian vein catheterization in the ipsilateral tilt position.

For last decades, the use of ultrasonography during vascular procedures has been suggested as a standard method that enhances overall success and reduces procedure-related complications, by professional organizations [9–11]. Ultrasonographic assessment can be applied before, during, and after central venous cannulation, and provides clinicians with advantages of success, speed, and safety, by visualizing vessel viability, size, and patency, as well as the location of other adjacent anatomically important structures [12, 33]. With respect to ultrasound-guided subclavian vein catheterization, although the quality of evidence is generally weak, the use of ultrasound-guidance is recommended in adult patients [9]. The results of the present study also provide supplemental evidence for the current guidelines. Especially, the primary venipuncture success rate was relatively low in this study. This can be overcome by the use of ultrasonography during catheterization. Previous studies demonstrated a significant increase in the first-pass success rate of subclavian vein catheterization and decrease in insertion attempts needed for venipuncture when ultrasound was used than landmark approach [34, 35].

### Limitations

This study had several limitations. First, there was a little anatomical discrepancy between the site which the csSCV was measured in stage I and the site which the actual venous puncture was achieved in stage II. Second, the operator performing catheterization could not be completely blinded to the position of the patients; this may have influenced the results. Third, although the operators had considerable experience with subclavian vein catheterization, they were more familiar with catheterization in patients placed in the supine position. This bias may have also influenced the overall success rate. Fourth, all catheterization was performed by two experienced practitioners. Therefore, our results may not be extrapolated to those with little experience. Fifth, subclavian vein catheterization was performed using the landmark technique. Therefore, it is hard to generalize our findings to ultrasound-guided catheterization, which is widely used during central venous catheterization. In addition, patients where there was a difficulty in locating the surface anatomical landmarks, such as morbid obesity, trauma, and chest wall deformity, were excluded from this study. The use of ultrasonography is also helpful for successful subclavian vein catheterization in such patients. Finally, this study was conducted in euvolemic patients. Therefore, this study does not clarify how enlargement of the vein in abnormal situations (i.e., hypovolemia) would affect successful catheter placement. Further studies are required to evaluate these relationships.

## Conclusions

The ipsilateral tilt position increased the csSCV significantly compared with the supine position. However, there was no corresponding effect on either the primary venipuncture success rate or clinical performance during the subclavian vein catheterization. Considering that additional efforts are required to maintain the head position of patients and to ensure patient safety during catheterization in the ipsilateral tilt position, we suggest the use of the supine position during landmark-based subclavian vein catheterization.

## Data Availability

The datasets supporting the conclusions of this article are included within the article.

## Abbreviation

csSCV: Cross-sectional area of the subclavian vein
CONSORT: Consolidated Standards of Reporting Trials
ICP: Intracranial pressure
